# A rare case of duodenal angiodysplasia—a case report

**DOI:** 10.1093/jscr/rjad138

**Published:** 2023-04-13

**Authors:** Saad Abdul Razzak, Faisal Nazir Awan, Amy Edward Murphy, Hamid Mustafa, Umer Mehmood, Jawad Ashraf

**Affiliations:** General Surgery Department, St. Luke’s General Hospital, Kilkenny, Ireland; General Surgery Department, St. Luke’s General Hospital, Kilkenny, Ireland; General Surgery Department, St. Luke’s General Hospital, Kilkenny, Ireland; General Surgery Department, St. Luke’s General Hospital, Kilkenny, Ireland; General Surgery Department, St. Luke’s General Hospital, Kilkenny, Ireland; General Surgery Department, St. Luke’s General Hospital, Kilkenny, Ireland

**Keywords:** upper gastrointestinal bleeding (UGIB), intestinal angiodysplasia, emergency embolization, operative intervention in GI bleed

## Abstract

Gastrointestinal bleeding can be manifested as a variety of symptoms and, often, it is difficult to classify as upper or lower gastrointestinal bleeding on mere symptomatology. This is a case report of a similar kind of patient who initially was diagnosed with fresh per rectum bleeding, subsequently diagnosed as bleeding angiodysplasia in duodenal diverticulum by a series of investigations and management including urgent oesophageal-gastroduodenoscopy (OGD), laparotomy, followed by computerized tomography-angiogram. As diagnosis was established after laparotomy, the patient was kept intubated and IR selective embolization was performed. Keeping this case report in view, it can be suggested that bleeding vascular malformation in any part duodenum should be considered as a cause of massive upper GI bleeding. Furthermore, if operative intervention is indicated, it should be preceded by OGD, not only for a therapeutic purpose but also as an adjunct for guidance for the operative plan.

## INTRODUCTION

An upper gastrointestinal bleed (UGIB) is defined as bleeding from the gastrointestinal tract proximal to the Ligament of Treitz. The presenting symptom of UGIB is hematemesis in 40–50% of the patients and melena in 90%. The presenting complaint is haematochezia in >90% of patients with a massive UGIB. Lower gastrointestinal bleeding (LGIB) can be present with melena or fresh per rectum (PR) bleeding similar in appearance to hematochezia. It can be difficult to differentiate between upper and lower GI bleeding by clinical history alone.

The initial management of a gastrointestinal bleed (GIB) is resuscitation and targeted transfusion therapy. Investigations of GIB include endoscopy, computerized tomography (CT) and CT angiography. Initial intervention is often at the time of endoscopy. For example, banding of oesophageal varices during an oesophageal-gastroduodenoscopy (OGD). There is potential for interventional radiology to perform selective angioembolization for the management of GIB where the patient has not responded to medical management and there is no safe endoscopic option. Lastly, operative intervention may be warranted in hemodynamically unstable patients where conservative and medical management have failed. Herein, we report the case of a rare cause of UGIB requiring significant investigation and intervention to manage.

## CASE PRESENTATION

We present the case of a 75-year-old male who was diagnosed with a single episode of bleeding PR. He had no prior history of GIB or gastrointestinal symptoms. Upon clinical examination, the patient was vitally stable. Examination of the gastrointestinal system demonstrated fresh blood on the digital rectal exam only. From initial laboratory results, the haemoglobin level was 9.5 mg/dl (13–14 mg/dl).

The patient was admitted to an acute surgical service for the management of a suspected GI bleed. He received initial resuscitation measures. CT scan of the abdomen and pelvis demonstrated diverticular disease of the sigmoid colon and a duodenal diverticulum at the duodenojejunal junction. There was no active source of bleeding at this time on imaging.

The patient remained in hospital for a 24-hour observation and repeat laboratory haemoglobin levels, given his initial presentation. During this observation period, the patient experienced large quantities of haematemesis with further fresh blood PR. He became vitally unstable with altered mental status, hypotension and tachycardia. A repeat haemoglobin level was 7.5 mg/dl.

The patient was transferred to an intensive care setting for invasive blood pressure monitoring, appropriate resuscitation with haematology guided transfusion therapy. The patient did not respond to this medical management. He was transferred to the theatre for an emergency OGD. This was performed under general anaesthetic. Endoscopy demonstrated large amounts of fresh blood in the stomach and duodenum. Despite adequate lavage and suctioning, the rate of fresh blood upon endoscopy prevented diagnostic views of the duodenum. The stomach was satisfactorily assessed and there was no source of bleed in the stomach. The patient proceeded to an upper midline laparotomy for ongoing, unstable GIB suspected to be originating from the duodenum. A longitudinal duodenotomy on the first part of the duodenum was performed. Large amounts of clots and fresh blood were found without a clear source. Duodenotomy was closed transversely as the source of bleeding was deemed to be distal to the anatomy accessible surgically. The laparotomy was closed in layers and the patient proceeded to radiology for a CT-angiogram (CTA).

**Figure 1:**
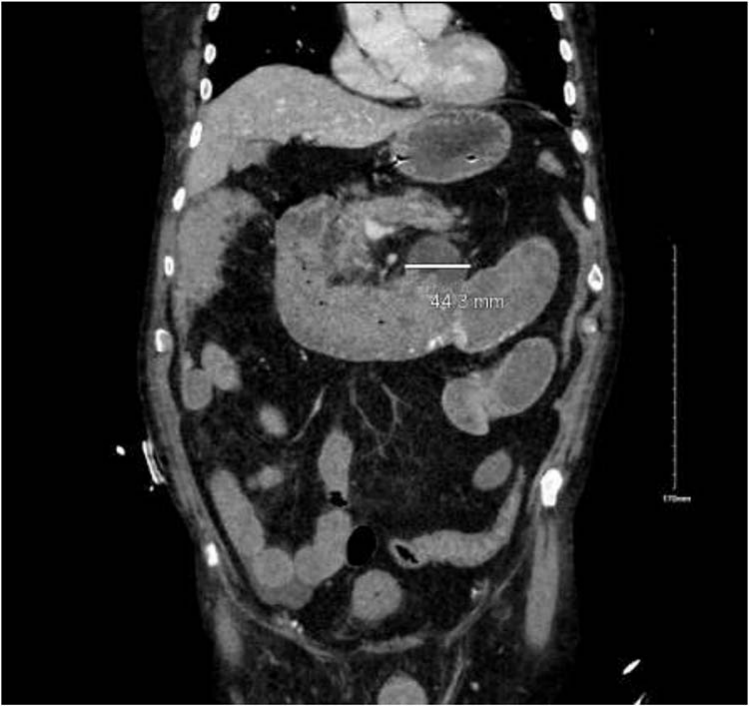
Duodenal diverticulum.

**Figure 2:**
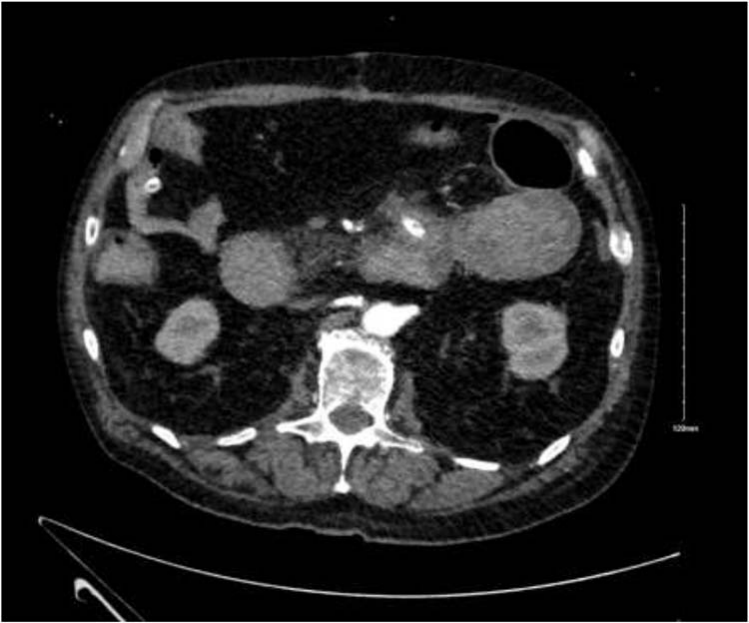
Angiodysplasia in duodenal diverticulum.

CTA demonstrated angiodysplasia in the duodenal diverticulum at the duodenojejunal junction with associated contrast blush (Figs 1 and 2). The urgent opinion of an interventional radiologist was sought in a tertiary centre. The patient was accepted for urgent embolization in an attempt to stabilize the patient. The patient required an intra-hospital transfer overnight.

The on-call interventional radiology team performed a successful embolization. A 6-French Sheath was passed through the left common femoral artery, and a 5-French Sim-1 catheter was advanced into the coeliac and common hepatic artery. A 2.4 French Direxon microcatheter was advanced into a branch of the superior mesenteric artery at the site of bleeding. Successful coil embolization with a 2 mm × 2 mm concerto micro coil was performed. A satisfactory angiographic result with DYNA-CT, also known as modern C-arm CT or cone-beam CTA, showed no extravasation.

**Figure 3:**
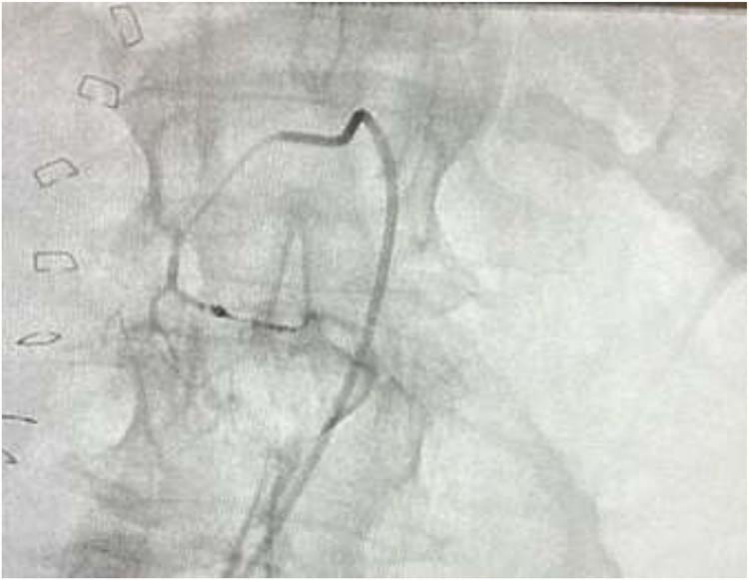
Contrast blush.

**Figure 4:**
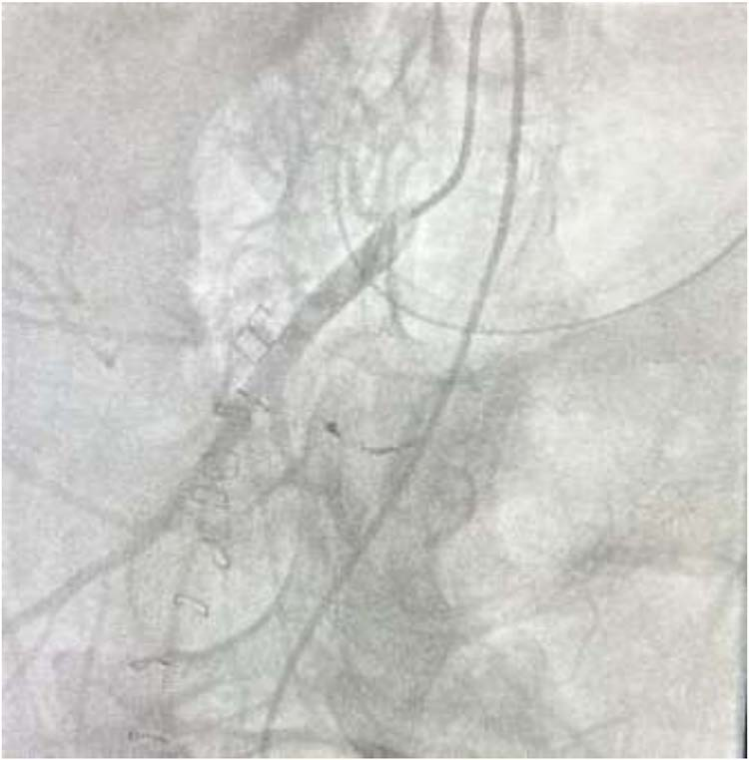
Post-embolization no contrast leak.

There were no immediate complications to the procedure. The patient returned to intensive care post-operatively. He remained intubated and vital laboratory tests were stabilized. He experienced acute renal failure post-procedure that required haemodialysis and prolonged intubation (Figs 3 and 4).

## DISCUSSION

Causes of UGIB can be broadly divided into variceal and non-variceal bleeding. The aetiology of non-variceal UGIB includes a duodenal ulcer, Mallory Weise tear, erosive gastritis, reflux oesophagitis and vascular malformation [1].

### Vascular malformations

Vascular malformation in the gastrointestinal tract can be categorized as hereditary and acquired hereditary hemorrhagic telangiectasia. Acquired causes include angiodysplasia, gastric antral vascular ectasia, radiation-induced vascular ectasia and Dieulafoy’s lesions.

### Arteriography

Arteriography can play an essential role in the diagnosis of angiodysplasia with high sensitivity and low false-negative results. However, due to the wide availability of a multidetector CT scan with contrast angiography, the diagnosis of vascular malformations may be made easily without formal angiography [2, 3].

### Management

The management of UGIB in clinical practice demonstrates variation in the timing of endoscopic intervention depending on patient and hospital factors. When variceal bleeding is suspected, it is generally recommended to perform an endoscopy as soon as the patient is hemodynamically stable [4]. The American Association for the Study of Liver Disease recommends endoscopy to be performed within 12 hours of the variceal bleed presentation [5, 9].

In the case of non-variceal bleeding such as our patient, the Asia-Pacific Working Group consensus and the European Society of Gastrointestinal Endoscopy, in 2018, recommend endoscopy within 24 hours of diagnosed [6, 7]. A patient undergoing emergency endoscopy has a 5-fold increased risk of adverse events including rebleeding, surgery, radiological intervention, repeated endoscopic management or even death [8].

Despite the advancement in endoscopic management of UGIB, surgery is an important treatment option demonstrated in our case. The literature demonstrates an increase in morbidity and mortality in patients who lose more than 6 units of blood or elderly patients with significant comorbidities [10]. Emergency transcatheter arterial embolization is a safe and standardized treatment option for the management of massive gastrointestinal arterial haemorrhage in selected patients, as seen in our case [11]. However, it is a service limited to tertiary centres and requires CTA imaging prior to establishing if the patient is a candidate for embolization.

In our patient, we proceeded with emergent endoscopy in a patient not responding to transfusion and intensive resuscitation. The patient proceeded to laparotomy directly from endoscopy as no bleeding source could be identified. Provided the hemodynamic stability, the CTA would have been done prior to laparotomy, and the source of bleeding would have been identified and managed with IR.

## CONCLUSION

Vascular malformation in the duodenum should be considered as a cause of massive upper GI bleeding, as demonstrated in our patient. Furthermore, if operative intervention is indicated, it should be preceded with OGD, not only for a therapeutic purpose but also as an adjunct for guidance for the operative plan.
